# 15α,20β-Dihydr­oxy-6β-meth­oxy-6,7-seco-6,20-ep­oxy-1,7-olide-*ent*-kaur-16-ene

**DOI:** 10.1107/S1600536810010573

**Published:** 2010-03-27

**Authors:** Fu-Lin Yan, He-Qin Zhan, Chuang Feng, Xue-Mei Di

**Affiliations:** a School of Pharmacy, Xinxiang Medical University, Xinxiang, Henan 453003, People’s Republic of China

## Abstract

The title compound, C_21_H_30_O_6_, a natural *ent*-kaurane diterpenoid, was obtained from the medicinal plant *Isodon serra*. The five rings in the mol­ecule exhibit the expected *cis* and *trans* junctions. The three six-membered rings adopt chair, twist-boat and boat conformations, while two five-membered rings adopt envelope conformations. There are two mol­ecules in the asymmetric unit, related by a non-crystallographic twofold screw axis; the main difference is in the different degrees of distortion of ring *B*. In the crystal, the mol­ecules are linked by inter­molecular O—H⋯O hydrogen bonds, forming chains along the *b* axis.

## Related literature

For the genus *Isodon* and diterpenoids, see: Sun *et al.* (2001 ▶); Yan *et al.* (2007 ▶, 2008 ▶). For bond-length data, see: Allen *et al.* (1987 ▶). For the structure of another *ent*-kaur-16-ene from an *Isodon* genus, see: Feng *et al.* (2010 ▶).
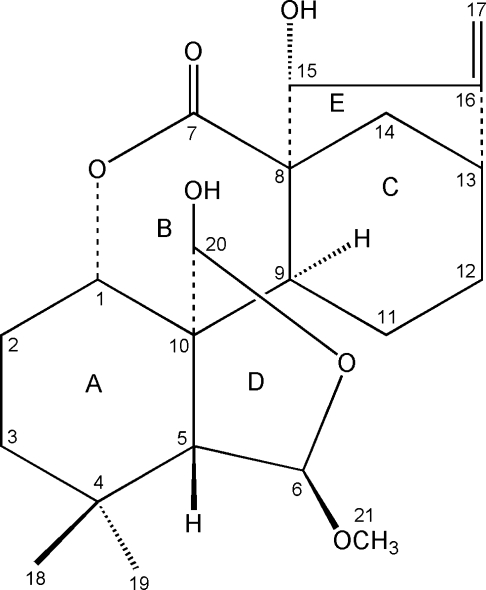


## Experimental

### 

#### Crystal data

C_21_H_30_O_6_
*M* *_r_* = 378.45
Monoclinic, 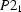

*a* = 13.145 (3) Å
*b* = 10.787 (2) Å
*c* = 14.074 (3) Å
β = 105.553 (3)°
*V* = 1922.6 (7) Å^3^
*Z* = 4
Mo *K*α radiation
μ = 0.10 mm^−1^
*T* = 93 K
0.60 × 0.55 × 0.55 mm

#### Data collection

Rigaku AFC10/Saturn724+ diffractometer
15542 measured reflections
4603 independent reflections
4328 reflections with *I* > 2σ(*I*)
*R*_int_ = 0.030

#### Refinement

*R*[*F* ^2^ > 2σ(*F* ^2^)] = 0.032
*wR*(*F* ^2^) = 0.079
*S* = 1.00
4603 reflections
509 parameters
1 restraint
H atoms treated by a mixture of independent and constrained refinement
Δρ_max_ = 0.28 e Å^−3^
Δρ_min_ = −0.18 e Å^−3^

### 

Data collection: *CrystalClear* (Rigaku/MSC, 2008 ▶); cell refinement: *CrystalClear*; data reduction: *CrystalClear*; program(s) used to solve structure: *SHELXS97* (Sheldrick, 2008 ▶); program(s) used to refine structure: *SHELXL97* (Sheldrick, 2008 ▶); molecular graphics: *SHELXTL* (Sheldrick, 2008 ▶); software used to prepare material for publication: *SHELXL97*.

## Supplementary Material

Crystal structure: contains datablocks global, I. DOI: 10.1107/S1600536810010573/zs2031sup1.cif

Structure factors: contains datablocks I. DOI: 10.1107/S1600536810010573/zs2031Isup2.hkl

Additional supplementary materials:  crystallographic information; 3D view; checkCIF report

## Figures and Tables

**Table 1:** Hydrogen-bond geometry (Å, °)

*D*—H⋯*A*	*D*—H	H⋯*A*	*D*⋯*A*	*D*—H⋯*A*
O3—H30⋯O5	0.85 (3)	1.88 (3)	2.695 (2)	161 (2)
O3′—H30′⋯O5′	0.88 (3)	2.01 (3)	2.806 (2)	150 (3)
O5—H50⋯O4′^i^	0.83 (3)	1.84 (3)	2.663 (2)	169 (3)
O5′—H50′⋯O1′^ii^	0.90 (3)	1.89 (3)	2.7663 (19)	165 (3)

Symmetry codes: (i) 
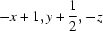
; (ii) 
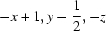
.

**Fig. 1.**
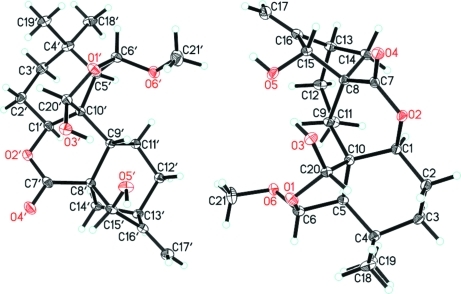
Molecular configuration and atom numbering scheme for the two independent molecules of (I) in the asymmetric unit. Displacement ellipsoids are drawn at the 50% probability level.

**Fig. 2.**
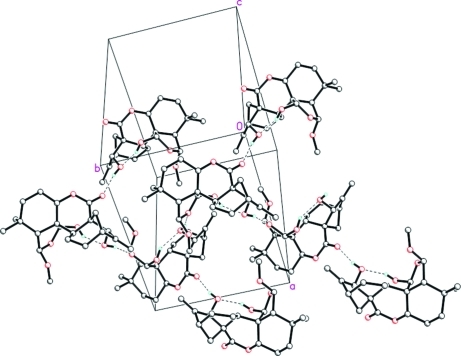
The crystal packing of (I), viewed along the c axis of the unit cell, showing the O—H···O hydrogen bonds as dashed lines.

## supplementary crystallographic information

### Comment

The title compound, 15α,20β-dihydroxy-6β-methoxy-6,7-seco-6,20- epoxy-1,7-olide-*ent*-kaur-16-ene, C_21_H_30_O_6_ (I) is a natural *ent*-kaurane diterpenoid isolated from the medicinal plant *Isodon serra* which is widely distributed in China. The plant material is used for the treatment of acute jaundice, hepatitis and acute cholecystitis in folk medicine. We extracted the leaves of *Isodon serra*, collected in the Henan province of China and obtained the title compound, and its structure was postulated from spectroscopic methods (Yan *et al.*, 2007). The X-ray crystallographic analysis confirms this proposed molecular structure (Fig. 1) and represents a second crystallographically characterized *ent*-kaur-16-ene compound isolated from *Isodon* plant material. The asymmetric unit of (I) contains two independent but similar molecules. In these there is a *trans* junction between ring *A* (C1—C5/C10) and ring *B* (C7—C10/C1/O2); *cis* junctions are present between ring *B* and ring *C* (C8/C9/C11—C14), ring *C* and ring *E* (C8/C13—C16), and ring *A* and ring *D* (C5/C6/O1/C20/C10). Ring *A* adopts a chair conformation, with an average torsion angle of 50.02 (2) °. Ring *C* adopts a boat conformation, while ring *B* adopts a twist boat conformation because of the presence of a carbonyl group. Ring *D* and ring *E* show envelope conformations. The two hydroxyl groups at C15 and C20 adopt α and β-orientations respectively, while the methoxy group at C6 adopts a β-orientation. Bond lengths and angles are within expected ranges (Allen *et al.*, 1987), with average values (Å) in the first molecule: C*sp*^3^—C*sp*^3^ = 1.543 (3), C*sp*^3^—C*sp*^2^ = 1.524 (3), C*sp*^2^—C*sp*^2^ (CC) = 1.326 (2), C*sp*^3^—O = 1.424 (2), and C*sp*^3^—C*sp*^3^ = 1.545 (3), C*sp*^3^—C*sp*^2^ = 1.525 (3), C*sp*^2^—C*sp*^2^ (CC) = 1.324 (2), C*sp*^3^—O = 1.448 (2) in the second molecule.

Compound (I) contains eight chiral centers at C1(*S*), C5(*R*), C8(*S*), C9(*S*), C10(*R*), C13(*R*), C15(*R*), and C20(*S*). Although the absolute configuration could not be reliably determined from anomalous dispersion effects, the negative optical rotation shows this compound to be of the *ent*-kaurane series as reported for the genus *Isodon* (Sun *et al.*, 2001), rather than of the kaurane series, allowing us to assign the correct configuration. In the crystal structure, intermolecular O—H···O hydrogen bonds (Table 1) are effective in the stabilization of the structure and are responsible for the formation of one-dimensional chains extending down the *b* axis of the unit cell (Fig. 2).

### Experimental

The dried and crushed leaves of *Isodon serra* (11 kg), collected from Henan Province, China, were extracted four times with Me_2_CO/H_2_O (7:3, *v*/*v*) at room temperature over a period of six days. The combined extract was filtered and the solvent was removed under reduced pressure. The extract was suspended in water and then partitioned successively with AcOEt, then concentrated to obtain a residue, which was then subjected to column chromatography over silica gel. Recrystallization from CHCl_3_/CH_3_OH (10:1), gave 55 mg of the title compound (m.p. 469-470 K; optical rotation: [α]_D_^23^ -141.0° (c 0.19, CH_3_OH). Crystals suitable for X-ray analysis were obtained by slow evaporation of a solution in CH_3_OH at room temperature.

### Refinement

All H atoms were included in calculated positions and refined as riding atoms, with C—H = 0.98Å (CH_3_), 0.99Å (CH_2_), and 1.00Å (CH), and with *U*_iso_(H) = 1.2 *U*_eq_(C). In the absence of significant anomalous scattering effects, Friedel pairs were merged. The choice of the enantiomer was based on comparison of the optical rotation with that of related compounds having known stereochemistry.

### Crystal data

**Table:**

C_21_H_30_O_6_	*F*(000) = 816
*M**_r_* = 378.45	*D*_x_ = 1.308 Mg m^−^^3^
Monoclinic, *P*2_1_	Melting point = 479–470 K
Hall symbol: P 2yb	Mo *K*α radiation, λ = 0.71073 Å
*a* = 13.145 (3) Å	Cell parameters from 6766 reflections
*b* = 10.787 (2) Å	θ = 3.0–27.5°
*c* = 14.074 (3) Å	µ = 0.10 mm^−^^1^
β = 105.553 (3)°	*T* = 93 K
*V* = 1922.6 (7) Å^3^	Block, colorless
*Z* = 4	0.60 × 0.55 × 0.55 mm

### Data collection

**Table:**

Rigaku AFC10/Saturn724+ diffractometer	4328 reflections with *I* > 2σ(*I*)
Radiation source: rotating anode	*R*_int_ = 0.030
graphite	θ_max_ = 27.5°, θ_min_ = 3.0°
Detector resolution: 28.5714 pixels mm^-1^	*h* = −17→13
multi–scan	*k* = −13→11
15542 measured reflections	*l* = −18→18
4603 independent reflections	

### Refinement

**Table:**

Refinement on *F*^2^	Primary atom site location: structure-invariant direct methods
Least-squares matrix: full	Secondary atom site location: difference Fourier map
*R*[*F*^2^ > 2σ(*F*^2^)] = 0.032	Hydrogen site location: inferred from neighbouring sites
*wR*(*F*^2^) = 0.079	H atoms treated by a mixture of independent and constrained refinement
*S* = 1.00	*w* = 1/[σ^2^(*F*_o_^2^) + (0.048*P*)^2^ + 0.166*P*] where *P* = (*F*_o_^2^ + 2*F*_c_^2^)/3
4603 reflections	(Δ/σ)_max_ = 0.001
509 parameters	Δρ_max_ = 0.28 e Å^−^^3^
1 restraint	Δρ_min_ = −0.18 e Å^−^^3^

### Special details

**Table:**

Geometry. All esds (except the esd in the dihedral angle between two l.s. planes) are estimated using the full covariance matrix. The cell esds are taken into account individually in the estimation of esds in distances, angles and torsion angles; correlations between esds in cell parameters are only used when they are defined by crystal symmetry. An approximate (isotropic) treatment of cell esds is used for estimating esds involving l.s. planes.
Refinement. Refinement of *F*^2^ against ALL reflections. The weighted *R*-factor *wR* and goodness of fit *S* are based on *F*^2^, conventional *R*-factors *R* are based on *F*, with *F* set to zero for negative *F*^2^. The threshold expression of *F*^2^ >σ(*F*^2^) is used only for calculating *R*-factors(gt) *etc*. and is not relevant to the choice of reflections for refinement. *R*-factors based on *F*^2^ are statistically about twice as large as those based on *F*, and *R*-factors based on ALL data will be even larger.

### Fractional atomic coordinates and isotropic or equivalent isotropic displacement parameters (Å^2^)

**Table:**

	*x*	*y*	*z*	*U*_iso_*/*U*_eq_	
O1	0.21535 (10)	0.59129 (13)	0.25270 (9)	0.0183 (3)	
O2	0.04936 (10)	0.81341 (13)	0.41597 (10)	0.0167 (3)	
O3	0.10251 (11)	0.76108 (13)	0.22402 (9)	0.0176 (3)	
O4	0.01659 (10)	0.98946 (13)	0.33514 (9)	0.0175 (3)	
O5	0.22703 (11)	0.96108 (14)	0.23065 (10)	0.0202 (3)	
O6	0.39423 (10)	0.61812 (13)	0.33706 (9)	0.0176 (3)	
C1	0.12818 (14)	0.72142 (18)	0.46303 (13)	0.0155 (4)	
H1	0.1716	0.7572	0.5265	0.019*	
C2	0.06994 (15)	0.61002 (19)	0.48695 (15)	0.0206 (4)	
H2A	0.0206	0.5776	0.4260	0.025*	
H2B	0.0286	0.6338	0.5334	0.025*	
C3	0.15069 (15)	0.51062 (19)	0.53337 (15)	0.0209 (4)	
H3A	0.1138	0.4400	0.5544	0.025*	
H3B	0.2010	0.5452	0.5929	0.025*	
C4	0.21236 (15)	0.46348 (19)	0.46194 (14)	0.0181 (4)	
C5	0.26792 (14)	0.57554 (17)	0.42642 (13)	0.0146 (4)	
H5	0.3327	0.5967	0.4799	0.018*	
C6	0.30092 (14)	0.55038 (18)	0.33222 (13)	0.0161 (4)	
H6	0.3136	0.4597	0.3257	0.019*	
C7	0.08214 (14)	0.91506 (18)	0.37685 (12)	0.0140 (4)	
C8	0.20072 (14)	0.93549 (18)	0.39484 (13)	0.0139 (4)	
C9	0.26956 (14)	0.81465 (18)	0.40115 (13)	0.0134 (3)	
H9	0.2969	0.8135	0.3414	0.016*	
C10	0.20151 (13)	0.69580 (17)	0.39725 (13)	0.0135 (4)	
C11	0.36637 (14)	0.81940 (18)	0.49186 (13)	0.0162 (4)	
H11A	0.3429	0.8026	0.5519	0.019*	
H11B	0.4162	0.7529	0.4859	0.019*	
C12	0.42462 (15)	0.94401 (19)	0.50428 (15)	0.0207 (4)	
H12A	0.4722	0.9458	0.4604	0.025*	
H12B	0.4688	0.9514	0.5731	0.025*	
C13	0.34882 (15)	1.05648 (18)	0.48017 (14)	0.0181 (4)	
H13	0.3769	1.1288	0.5238	0.022*	
C14	0.23949 (15)	1.01650 (19)	0.48929 (13)	0.0171 (4)	
H14A	0.1927	1.0887	0.4882	0.021*	
H14B	0.2449	0.9676	0.5500	0.021*	
C15	0.22230 (14)	1.02618 (18)	0.31723 (13)	0.0152 (4)	
H15	0.1639	1.0885	0.2996	0.018*	
C16	0.32415 (14)	1.09148 (19)	0.37185 (14)	0.0173 (4)	
C17	0.37885 (16)	1.1670 (2)	0.32979 (15)	0.0233 (4)	
H17A	0.4410	1.2058	0.3686	0.028*	
H17B	0.3558	1.1822	0.2608	0.028*	
C18	0.29977 (15)	0.37646 (19)	0.52032 (15)	0.0203 (4)	
H18A	0.3434	0.4205	0.5778	0.024*	
H18B	0.3439	0.3498	0.4780	0.024*	
H18C	0.2676	0.3037	0.5423	0.024*	
C19	0.13931 (16)	0.38538 (19)	0.37930 (16)	0.0234 (4)	
H19A	0.1130	0.3138	0.4084	0.028*	
H19B	0.1790	0.3566	0.3337	0.028*	
H19C	0.0796	0.4363	0.3434	0.028*	
C20	0.14130 (14)	0.66459 (18)	0.28865 (13)	0.0146 (4)	
H20	0.0804	0.6098	0.2904	0.018*	
C21	0.43104 (16)	0.6056 (2)	0.25089 (15)	0.0280 (5)	
H21A	0.4347	0.5175	0.2351	0.034*	
H21B	0.5014	0.6426	0.2630	0.034*	
H21C	0.3823	0.6480	0.1955	0.034*	
O1'	0.61754 (10)	1.02618 (13)	0.02824 (9)	0.0193 (3)	
O2'	0.83569 (11)	0.76955 (13)	−0.03698 (10)	0.0198 (3)	
O3'	0.60905 (11)	0.83925 (14)	−0.05461 (10)	0.0207 (3)	
O4'	0.74557 (12)	0.59810 (15)	−0.08204 (10)	0.0255 (3)	
O5'	0.56677 (11)	0.64907 (14)	0.06543 (10)	0.0214 (3)	
O6'	0.64390 (10)	1.01648 (14)	0.19995 (10)	0.0214 (3)	
C1'	0.86959 (15)	0.86529 (18)	0.03932 (14)	0.0171 (4)	
H1'	0.9228	0.8276	0.0965	0.021*	
C2'	0.92367 (15)	0.9662 (2)	−0.00343 (15)	0.0218 (4)	
H2C'	0.8754	0.9986	−0.0650	0.026*	
H2D'	0.9874	0.9328	−0.0191	0.026*	
C3'	0.95441 (15)	1.0701 (2)	0.07316 (15)	0.0226 (4)	
H3C'	0.9957	1.1335	0.0488	0.027*	
H3D'	1.0001	1.0355	0.1352	0.027*	
C4'	0.85739 (15)	1.13206 (19)	0.09477 (14)	0.0198 (4)	
C5'	0.79168 (14)	1.03008 (18)	0.13136 (13)	0.0153 (4)	
H5'	0.8270	1.0123	0.2023	0.018*	
C6'	0.67826 (14)	1.07021 (19)	0.12311 (13)	0.0176 (4)	
H6'	0.6739	1.1626	0.1262	0.021*	
C7'	0.77627 (15)	0.67684 (19)	−0.01898 (14)	0.0185 (4)	
C8'	0.75785 (14)	0.66697 (18)	0.08296 (13)	0.0140 (4)	
C9'	0.74751 (14)	0.79359 (17)	0.13467 (13)	0.0132 (4)	
H9'	0.6720	0.8026	0.1355	0.016*	
C10'	0.77399 (14)	0.90470 (18)	0.07427 (13)	0.0138 (4)	
C11'	0.81477 (14)	0.79157 (18)	0.24275 (13)	0.0152 (4)	
H11C	0.8902	0.8005	0.2445	0.018*	
H11D	0.7951	0.8630	0.2783	0.018*	
C12'	0.79969 (15)	0.67141 (19)	0.29529 (13)	0.0170 (4)	
H12C	0.7301	0.6738	0.3099	0.020*	
H12D	0.8547	0.6664	0.3589	0.020*	
C13'	0.80568 (14)	0.55338 (18)	0.23381 (13)	0.0164 (4)	
H13'	0.8473	0.4859	0.2753	0.020*	
C14'	0.85067 (14)	0.58773 (19)	0.14703 (13)	0.0167 (4)	
H14C	0.8642	0.5132	0.1112	0.020*	
H14D	0.9164	0.6368	0.1691	0.020*	
C15'	0.66278 (14)	0.57987 (19)	0.08060 (14)	0.0168 (4)	
H15'	0.6552	0.5190	0.0255	0.020*	
C16'	0.69535 (14)	0.51043 (19)	0.17910 (14)	0.0173 (4)	
C17'	0.63873 (16)	0.4233 (2)	0.20734 (15)	0.0227 (4)	
H17C	0.6667	0.3805	0.2677	0.027*	
H17D	0.5702	0.4037	0.1673	0.027*	
C18'	0.89630 (17)	1.2245 (2)	0.18054 (16)	0.0249 (4)	
H18D	0.9406	1.1808	0.2379	0.030*	
H18E	0.8354	1.2611	0.1979	0.030*	
H18F	0.9376	1.2902	0.1603	0.030*	
C19'	0.79649 (17)	1.2062 (2)	0.00367 (16)	0.0244 (4)	
H19D	0.7352	1.2464	0.0177	0.029*	
H19E	0.7725	1.1500	−0.0526	0.029*	
H19F	0.8430	1.2695	−0.0121	0.029*	
C20'	0.67574 (14)	0.93572 (18)	−0.01246 (13)	0.0164 (4)	
H20'	0.7009	0.9770	−0.0656	0.020*	
C21'	0.53768 (18)	1.0486 (3)	0.19681 (18)	0.0343 (6)	
H21D	0.5290	1.1387	0.1909	0.041*	
H21E	0.5218	1.0205	0.2575	0.041*	
H21F	0.4893	1.0085	0.1399	0.041*	
H30	0.1504 (19)	0.812 (2)	0.2211 (17)	0.022 (6)*	
H50	0.2315 (19)	1.011 (3)	0.1869 (19)	0.028 (7)*	
H30'	0.586 (2)	0.803 (3)	−0.009 (2)	0.043 (8)*	
H50'	0.514 (2)	0.598 (3)	0.035 (2)	0.049 (9)*	

### Atomic displacement parameters (Å^2^)

**Table:**

	*U*^11^	*U*^22^	*U*^33^	*U*^12^	*U*^13^	*U*^23^
O1	0.0177 (6)	0.0204 (7)	0.0149 (6)	0.0048 (6)	0.0010 (5)	−0.0037 (5)
O2	0.0141 (6)	0.0168 (7)	0.0210 (6)	0.0026 (5)	0.0077 (5)	0.0051 (5)
O3	0.0163 (6)	0.0180 (7)	0.0164 (6)	−0.0010 (6)	0.0009 (5)	0.0029 (5)
O4	0.0153 (6)	0.0179 (7)	0.0188 (6)	0.0032 (5)	0.0036 (5)	0.0025 (5)
O5	0.0291 (8)	0.0181 (8)	0.0136 (6)	−0.0051 (6)	0.0064 (6)	0.0012 (6)
O6	0.0161 (6)	0.0206 (8)	0.0174 (6)	0.0010 (5)	0.0067 (5)	−0.0003 (5)
C1	0.0138 (8)	0.0164 (9)	0.0167 (8)	0.0036 (7)	0.0049 (7)	0.0036 (7)
C2	0.0182 (9)	0.0196 (11)	0.0268 (10)	0.0031 (8)	0.0107 (8)	0.0068 (8)
C3	0.0189 (9)	0.0185 (11)	0.0269 (10)	0.0019 (8)	0.0088 (8)	0.0088 (8)
C4	0.0168 (9)	0.0153 (10)	0.0222 (9)	0.0002 (7)	0.0052 (7)	0.0041 (7)
C5	0.0141 (8)	0.0128 (10)	0.0160 (8)	0.0015 (7)	0.0025 (7)	−0.0002 (7)
C6	0.0142 (8)	0.0153 (10)	0.0172 (8)	0.0015 (7)	0.0017 (7)	0.0000 (7)
C7	0.0155 (8)	0.0149 (10)	0.0122 (7)	0.0007 (7)	0.0047 (7)	−0.0011 (7)
C8	0.0150 (8)	0.0129 (9)	0.0132 (8)	0.0007 (7)	0.0029 (6)	0.0006 (7)
C9	0.0131 (8)	0.0127 (9)	0.0130 (7)	0.0023 (7)	0.0012 (6)	−0.0001 (7)
C10	0.0115 (8)	0.0145 (10)	0.0140 (8)	0.0009 (7)	0.0025 (7)	0.0013 (7)
C11	0.0133 (8)	0.0174 (10)	0.0158 (8)	0.0010 (7)	0.0002 (7)	−0.0004 (7)
C12	0.0157 (9)	0.0167 (10)	0.0254 (9)	−0.0012 (8)	−0.0018 (7)	−0.0022 (8)
C13	0.0180 (9)	0.0149 (10)	0.0192 (9)	0.0011 (7)	0.0013 (7)	−0.0029 (7)
C14	0.0192 (9)	0.0164 (10)	0.0144 (8)	0.0022 (8)	0.0023 (7)	−0.0023 (7)
C15	0.0156 (8)	0.0136 (9)	0.0158 (8)	0.0005 (7)	0.0033 (7)	0.0011 (7)
C16	0.0160 (8)	0.0157 (10)	0.0200 (9)	0.0008 (7)	0.0042 (7)	−0.0033 (7)
C17	0.0217 (9)	0.0251 (11)	0.0240 (9)	−0.0063 (9)	0.0076 (8)	−0.0045 (9)
C18	0.0200 (9)	0.0158 (10)	0.0253 (10)	0.0020 (8)	0.0066 (8)	0.0049 (8)
C19	0.0217 (10)	0.0146 (10)	0.0311 (11)	−0.0031 (8)	0.0024 (8)	0.0029 (8)
C20	0.0142 (8)	0.0133 (9)	0.0156 (8)	−0.0002 (7)	0.0024 (7)	0.0002 (7)
C21	0.0233 (10)	0.0427 (14)	0.0205 (9)	0.0012 (10)	0.0106 (8)	−0.0015 (9)
O1'	0.0156 (6)	0.0206 (7)	0.0193 (6)	0.0059 (6)	0.0008 (5)	−0.0019 (6)
O2'	0.0231 (7)	0.0197 (7)	0.0181 (6)	0.0019 (6)	0.0082 (5)	−0.0013 (5)
O3'	0.0186 (7)	0.0220 (8)	0.0183 (7)	0.0001 (6)	−0.0008 (5)	−0.0018 (6)
O4'	0.0321 (8)	0.0250 (8)	0.0194 (7)	0.0013 (7)	0.0070 (6)	−0.0073 (6)
O5'	0.0118 (6)	0.0189 (8)	0.0294 (7)	0.0001 (5)	−0.0014 (5)	−0.0013 (6)
O6'	0.0188 (7)	0.0240 (8)	0.0227 (7)	0.0052 (6)	0.0078 (5)	0.0011 (6)
C1'	0.0172 (9)	0.0176 (10)	0.0169 (9)	0.0024 (7)	0.0052 (7)	−0.0003 (7)
C2'	0.0193 (9)	0.0229 (11)	0.0251 (10)	0.0016 (8)	0.0095 (8)	0.0045 (8)
C3'	0.0167 (9)	0.0234 (11)	0.0275 (10)	−0.0025 (8)	0.0055 (8)	0.0067 (8)
C4'	0.0190 (9)	0.0158 (10)	0.0221 (9)	−0.0012 (8)	0.0013 (7)	0.0029 (8)
C5'	0.0149 (8)	0.0135 (9)	0.0163 (8)	0.0019 (7)	0.0021 (7)	−0.0003 (7)
C6'	0.0185 (9)	0.0161 (10)	0.0172 (9)	0.0027 (7)	0.0027 (7)	−0.0018 (7)
C7'	0.0179 (9)	0.0194 (10)	0.0184 (9)	0.0062 (8)	0.0050 (7)	−0.0023 (8)
C8'	0.0135 (8)	0.0132 (9)	0.0141 (8)	0.0015 (7)	0.0017 (6)	−0.0016 (7)
C9'	0.0133 (8)	0.0129 (9)	0.0121 (8)	0.0009 (7)	0.0013 (6)	−0.0011 (7)
C10'	0.0131 (8)	0.0139 (9)	0.0134 (8)	0.0010 (7)	0.0019 (7)	−0.0001 (7)
C11'	0.0154 (8)	0.0153 (10)	0.0127 (8)	−0.0004 (7)	0.0001 (7)	−0.0012 (7)
C12'	0.0172 (8)	0.0179 (10)	0.0142 (8)	0.0000 (7)	0.0015 (7)	−0.0016 (7)
C13'	0.0159 (8)	0.0146 (10)	0.0173 (8)	0.0001 (7)	0.0018 (7)	0.0014 (7)
C14'	0.0154 (8)	0.0147 (9)	0.0187 (9)	0.0036 (7)	0.0022 (7)	−0.0007 (7)
C15'	0.0147 (8)	0.0148 (10)	0.0192 (9)	0.0007 (7)	0.0015 (7)	−0.0037 (7)
C16'	0.0164 (8)	0.0157 (10)	0.0193 (9)	0.0015 (7)	0.0038 (7)	−0.0035 (7)
C17'	0.0206 (10)	0.0219 (11)	0.0243 (9)	−0.0014 (8)	0.0037 (8)	−0.0012 (8)
C18'	0.0254 (10)	0.0162 (10)	0.0287 (10)	−0.0025 (9)	−0.0005 (8)	0.0006 (8)
C19'	0.0235 (10)	0.0193 (11)	0.0284 (10)	0.0001 (8)	0.0033 (8)	0.0074 (8)
C20'	0.0151 (9)	0.0182 (10)	0.0146 (8)	0.0039 (7)	0.0019 (7)	0.0001 (7)
C21'	0.0265 (11)	0.0436 (15)	0.0377 (12)	0.0112 (10)	0.0173 (10)	0.0071 (11)

### Geometric parameters (Å, °)

**Table:**

O1—C6	1.427 (2)	O1'—C6'	1.440 (2)
O1—C20	1.447 (2)	O1'—C20'	1.449 (2)
O2—C7	1.348 (2)	O2'—C7'	1.334 (2)
O2—C1	1.459 (2)	O2'—C1'	1.470 (2)
O3—C20	1.386 (2)	O3'—C20'	1.387 (2)
O3—H30	0.85 (3)	O3'—H30'	0.88 (3)
O4—C7	1.208 (2)	O4'—C7'	1.216 (2)
O5—C15	1.422 (2)	O5'—C15'	1.432 (2)
O5—H50	0.83 (3)	O5'—H50'	0.90 (3)
O6—C6	1.414 (2)	O6'—C6'	1.404 (2)
O6—C21	1.428 (2)	O6'—C21'	1.428 (2)
C1—C2	1.511 (3)	C1'—C2'	1.510 (3)
C1—C10	1.530 (2)	C1'—C10'	1.528 (3)
C1—H1	1.0000	C1'—H1'	1.0000
C2—C3	1.527 (3)	C2'—C3'	1.532 (3)
C2—H2A	0.9900	C2'—H2C'	0.9900
C2—H2B	0.9900	C2'—H2D'	0.9900
C3—C4	1.537 (3)	C3'—C4'	1.541 (3)
C3—H3A	0.9900	C3'—H3C'	0.9900
C3—H3B	0.9900	C3'—H3D'	0.9900
C4—C18	1.540 (3)	C4'—C19'	1.541 (3)
C4—C19	1.545 (3)	C4'—C18'	1.543 (3)
C4—C5	1.562 (3)	C4'—C5'	1.568 (3)
C5—C6	1.526 (3)	C5'—C6'	1.527 (2)
C5—C10	1.556 (3)	C5'—C10'	1.558 (3)
C5—H5	1.0000	C5'—H5'	1.0000
C6—H6	1.0000	C6'—H6'	1.0000
C7—C8	1.527 (2)	C7'—C8'	1.521 (3)
C8—C15	1.548 (3)	C8'—C15'	1.557 (3)
C8—C14	1.558 (2)	C8'—C14'	1.563 (2)
C8—C9	1.576 (3)	C8'—C9'	1.571 (3)
C9—C11	1.543 (2)	C9'—C11'	1.541 (2)
C9—C10	1.556 (3)	C9'—C10'	1.562 (3)
C9—H9	1.0000	C9'—H9'	1.0000
C10—C20	1.558 (2)	C10'—C20'	1.557 (2)
C11—C12	1.534 (3)	C11'—C12'	1.531 (3)
C11—H11A	0.9900	C11'—H11C	0.9900
C11—H11B	0.9900	C11'—H11D	0.9900
C12—C13	1.549 (3)	C12'—C13'	1.553 (3)
C12—H12A	0.9900	C12'—H12C	0.9900
C12—H12B	0.9900	C12'—H12D	0.9900
C13—C16	1.519 (3)	C13'—C16'	1.521 (3)
C13—C14	1.538 (3)	C13'—C14'	1.538 (3)
C13—H13	1.0000	C13'—H13'	1.0000
C14—H14A	0.9900	C14'—H14C	0.9900
C14—H14B	0.9900	C14'—H14D	0.9900
C15—C16	1.526 (3)	C15'—C16'	1.532 (3)
C15—H15	1.0000	C15'—H15'	1.0000
C16—C17	1.326 (3)	C16'—C17'	1.324 (3)
C17—H17A	0.9500	C17'—H17C	0.9500
C17—H17B	0.9500	C17'—H17D	0.9500
C18—H18A	0.9800	C18'—H18D	0.9800
C18—H18B	0.9800	C18'—H18E	0.9800
C18—H18C	0.9800	C18'—H18F	0.9800
C19—H19A	0.9800	C19'—H19D	0.9800
C19—H19B	0.9800	C19'—H19E	0.9800
C19—H19C	0.9800	C19'—H19F	0.9800
C20—H20	1.0000	C20'—H20'	1.0000
C21—H21A	0.9800	C21'—H21D	0.9800
C21—H21B	0.9800	C21'—H21E	0.9800
C21—H21C	0.9800	C21'—H21F	0.9800
			
C6—O1—C20	110.95 (13)	C6'—O1'—C20'	111.62 (13)
C7—O2—C1	118.20 (14)	C7'—O2'—C1'	117.78 (14)
C20—O3—H30	111.9 (16)	C20'—O3'—H30'	109 (2)
C15—O5—H50	110.0 (18)	C15'—O5'—H50'	107 (2)
C6—O6—C21	113.42 (15)	C6'—O6'—C21'	112.95 (15)
O2—C1—C2	107.55 (14)	O2'—C1'—C2'	107.35 (15)
O2—C1—C10	109.52 (14)	O2'—C1'—C10'	108.44 (15)
C2—C1—C10	115.49 (16)	C2'—C1'—C10'	116.32 (16)
O2—C1—H1	108.0	O2'—C1'—H1'	108.2
C2—C1—H1	108.0	C2'—C1'—H1'	108.2
C10—C1—H1	108.0	C10'—C1'—H1'	108.2
C1—C2—C3	108.56 (15)	C1'—C2'—C3'	108.08 (16)
C1—C2—H2A	110.0	C1'—C2'—H2C'	110.1
C3—C2—H2A	110.0	C3'—C2'—H2C'	110.1
C1—C2—H2B	110.0	C1'—C2'—H2D'	110.1
C3—C2—H2B	110.0	C3'—C2'—H2D'	110.1
H2A—C2—H2B	108.4	H2C'—C2'—H2D'	108.4
C2—C3—C4	112.37 (16)	C2'—C3'—C4'	112.34 (16)
C2—C3—H3A	109.1	C2'—C3'—H3C'	109.1
C4—C3—H3A	109.1	C4'—C3'—H3C'	109.1
C2—C3—H3B	109.1	C2'—C3'—H3D'	109.1
C4—C3—H3B	109.1	C4'—C3'—H3D'	109.1
H3A—C3—H3B	107.9	H3C'—C3'—H3D'	107.9
C3—C4—C18	107.56 (15)	C19'—C4'—C3'	109.51 (17)
C3—C4—C19	110.03 (16)	C19'—C4'—C18'	107.74 (17)
C18—C4—C19	107.07 (16)	C3'—C4'—C18'	108.45 (16)
C3—C4—C5	109.09 (16)	C19'—C4'—C5'	115.36 (16)
C18—C4—C5	107.25 (15)	C3'—C4'—C5'	108.56 (16)
C19—C4—C5	115.51 (16)	C18'—C4'—C5'	107.00 (16)
C6—C5—C10	100.90 (14)	C6'—C5'—C10'	101.50 (14)
C6—C5—C4	114.00 (15)	C6'—C5'—C4'	112.87 (15)
C10—C5—C4	116.88 (14)	C10'—C5'—C4'	117.29 (15)
C6—C5—H5	108.2	C6'—C5'—H5'	108.2
C10—C5—H5	108.2	C10'—C5'—H5'	108.2
C4—C5—H5	108.2	C4'—C5'—H5'	108.2
O6—C6—O1	111.67 (15)	O6'—C6'—O1'	111.42 (15)
O6—C6—C5	108.14 (15)	O6'—C6'—C5'	109.49 (15)
O1—C6—C5	106.17 (14)	O1'—C6'—C5'	105.31 (14)
O6—C6—H6	110.3	O6'—C6'—H6'	110.2
O1—C6—H6	110.3	O1'—C6'—H6'	110.2
C5—C6—H6	110.3	C5'—C6'—H6'	110.2
O4—C7—O2	118.31 (16)	O4'—C7'—O2'	118.78 (17)
O4—C7—C8	123.23 (17)	O4'—C7'—C8'	122.24 (18)
O2—C7—C8	118.26 (15)	O2'—C7'—C8'	118.74 (16)
C7—C8—C15	110.26 (14)	C7'—C8'—C15'	110.61 (15)
C7—C8—C14	107.96 (14)	C7'—C8'—C14'	106.58 (14)
C15—C8—C14	99.65 (15)	C15'—C8'—C14'	100.39 (15)
C7—C8—C9	115.81 (15)	C7'—C8'—C9'	115.56 (15)
C15—C8—C9	110.57 (14)	C15'—C8'—C9'	111.47 (15)
C14—C8—C9	111.37 (14)	C14'—C8'—C9'	111.07 (14)
C11—C9—C10	113.29 (15)	C11'—C9'—C10'	113.29 (15)
C11—C9—C8	110.67 (15)	C11'—C9'—C8'	110.31 (15)
C10—C9—C8	111.33 (14)	C10'—C9'—C8'	110.83 (14)
C11—C9—H9	107.1	C11'—C9'—H9'	107.4
C10—C9—H9	107.1	C10'—C9'—H9'	107.4
C8—C9—H9	107.1	C8'—C9'—H9'	107.4
C1—C10—C9	106.22 (15)	C1'—C10'—C20'	112.86 (15)
C1—C10—C5	113.00 (15)	C1'—C10'—C5'	112.69 (15)
C9—C10—C5	113.65 (14)	C20'—C10'—C5'	101.27 (14)
C1—C10—C20	113.33 (14)	C1'—C10'—C9'	106.52 (15)
C9—C10—C20	110.41 (14)	C20'—C10'—C9'	109.42 (15)
C5—C10—C20	100.38 (14)	C5'—C10'—C9'	114.18 (14)
C12—C11—C9	113.53 (16)	C12'—C11'—C9'	111.98 (15)
C12—C11—H11A	108.9	C12'—C11'—H11C	109.2
C9—C11—H11A	108.9	C9'—C11'—H11C	109.2
C12—C11—H11B	108.9	C12'—C11'—H11D	109.2
C9—C11—H11B	108.9	C9'—C11'—H11D	109.2
H11A—C11—H11B	107.7	H11C—C11'—H11D	107.9
C11—C12—C13	112.92 (15)	C11'—C12'—C13'	113.10 (14)
C11—C12—H12A	109.0	C11'—C12'—H12C	109.0
C13—C12—H12A	109.0	C13'—C12'—H12C	109.0
C11—C12—H12B	109.0	C11'—C12'—H12D	109.0
C13—C12—H12B	109.0	C13'—C12'—H12D	109.0
H12A—C12—H12B	107.8	H12C—C12'—H12D	107.8
C16—C13—C14	101.58 (14)	C16'—C13'—C14'	100.89 (14)
C16—C13—C12	111.49 (16)	C16'—C13'—C12'	110.36 (15)
C14—C13—C12	108.82 (16)	C14'—C13'—C12'	109.43 (15)
C16—C13—H13	111.5	C16'—C13'—H13'	111.9
C14—C13—H13	111.5	C14'—C13'—H13'	111.9
C12—C13—H13	111.5	C12'—C13'—H13'	111.9
C13—C14—C8	100.67 (14)	C13'—C14'—C8'	100.54 (14)
C13—C14—H14A	111.6	C13'—C14'—H14C	111.7
C8—C14—H14A	111.6	C8'—C14'—H14C	111.7
C13—C14—H14B	111.6	C13'—C14'—H14D	111.7
C8—C14—H14B	111.6	C8'—C14'—H14D	111.7
H14A—C14—H14B	109.4	H14C—C14'—H14D	109.4
O5—C15—C16	115.19 (15)	O5'—C15'—C16'	114.27 (15)
O5—C15—C8	110.54 (15)	O5'—C15'—C8'	111.03 (16)
C16—C15—C8	103.79 (14)	C16'—C15'—C8'	104.35 (14)
O5—C15—H15	109.0	O5'—C15'—H15'	109.0
C16—C15—H15	109.0	C16'—C15'—H15'	109.0
C8—C15—H15	109.0	C8'—C15'—H15'	109.0
C17—C16—C13	127.25 (18)	C17'—C16'—C13'	126.92 (18)
C17—C16—C15	124.48 (17)	C17'—C16'—C15'	125.12 (17)
C13—C16—C15	108.26 (15)	C13'—C16'—C15'	107.84 (15)
C16—C17—H17A	120.0	C16'—C17'—H17C	120.0
C16—C17—H17B	120.0	C16'—C17'—H17D	120.0
H17A—C17—H17B	120.0	H17C—C17'—H17D	120.0
C4—C18—H18A	109.5	C4'—C18'—H18D	109.5
C4—C18—H18B	109.5	C4'—C18'—H18E	109.5
H18A—C18—H18B	109.5	H18D—C18'—H18E	109.5
C4—C18—H18C	109.5	C4'—C18'—H18F	109.5
H18A—C18—H18C	109.5	H18D—C18'—H18F	109.5
H18B—C18—H18C	109.5	H18E—C18'—H18F	109.5
C4—C19—H19A	109.5	C4'—C19'—H19D	109.5
C4—C19—H19B	109.5	C4'—C19'—H19E	109.5
H19A—C19—H19B	109.5	H19D—C19'—H19E	109.5
C4—C19—H19C	109.5	C4'—C19'—H19F	109.5
H19A—C19—H19C	109.5	H19D—C19'—H19F	109.5
H19B—C19—H19C	109.5	H19E—C19'—H19F	109.5
O3—C20—O1	110.15 (14)	O3'—C20'—O1'	109.51 (14)
O3—C20—C10	118.82 (16)	O3'—C20'—C10'	118.05 (16)
O1—C20—C10	104.41 (13)	O1'—C20'—C10'	104.46 (14)
O3—C20—H20	107.7	O3'—C20'—H20'	108.1
O1—C20—H20	107.7	O1'—C20'—H20'	108.1
C10—C20—H20	107.7	C10'—C20'—H20'	108.1
O6—C21—H21A	109.5	O6'—C21'—H21D	109.5
O6—C21—H21B	109.5	O6'—C21'—H21E	109.5
H21A—C21—H21B	109.5	H21D—C21'—H21E	109.5
O6—C21—H21C	109.5	O6'—C21'—H21F	109.5
H21A—C21—H21C	109.5	H21D—C21'—H21F	109.5
H21B—C21—H21C	109.5	H21E—C21'—H21F	109.5
			
C7—O2—C1—C2	−171.93 (15)	C7'—O2'—C1'—C2'	−173.29 (15)
C7—O2—C1—C10	−45.7 (2)	C7'—O2'—C1'—C10'	−46.9 (2)
O2—C1—C2—C3	178.22 (15)	O2'—C1'—C2'—C3'	177.07 (15)
C10—C1—C2—C3	55.6 (2)	C10'—C1'—C2'—C3'	55.5 (2)
C1—C2—C3—C4	−63.5 (2)	C1'—C2'—C3'—C4'	−63.9 (2)
C2—C3—C4—C18	173.19 (16)	C2'—C3'—C4'—C19'	−69.0 (2)
C2—C3—C4—C19	−70.5 (2)	C2'—C3'—C4'—C18'	173.70 (16)
C2—C3—C4—C5	57.2 (2)	C2'—C3'—C4'—C5'	57.8 (2)
C3—C4—C5—C6	−161.07 (15)	C19'—C4'—C5'—C6'	−38.0 (2)
C18—C4—C5—C6	82.71 (19)	C3'—C4'—C5'—C6'	−161.32 (15)
C19—C4—C5—C6	−36.6 (2)	C18'—C4'—C5'—C6'	81.82 (18)
C3—C4—C5—C10	−43.8 (2)	C19'—C4'—C5'—C10'	79.4 (2)
C18—C4—C5—C10	−160.05 (15)	C3'—C4'—C5'—C10'	−43.9 (2)
C19—C4—C5—C10	80.7 (2)	C18'—C4'—C5'—C10'	−160.72 (15)
C21—O6—C6—O1	−61.4 (2)	C21'—O6'—C6'—O1'	−63.3 (2)
C21—O6—C6—C5	−177.86 (16)	C21'—O6'—C6'—C5'	−179.37 (17)
C20—O1—C6—O6	−105.03 (17)	C20'—O1'—C6'—O6'	−103.68 (17)
C20—O1—C6—C5	12.6 (2)	C20'—O1'—C6'—C5'	14.9 (2)
C10—C5—C6—O6	86.38 (16)	C10'—C5'—C6'—O6'	86.15 (17)
C4—C5—C6—O6	−147.48 (15)	C4'—C5'—C6'—O6'	−147.43 (16)
C10—C5—C6—O1	−33.58 (18)	C10'—C5'—C6'—O1'	−33.75 (18)
C4—C5—C6—O1	92.56 (18)	C4'—C5'—C6'—O1'	92.67 (18)
C1—O2—C7—O4	177.72 (16)	C1'—O2'—C7'—O4'	178.48 (17)
C1—O2—C7—C8	−7.2 (2)	C1'—O2'—C7'—C8'	−7.1 (2)
O4—C7—C8—C15	−25.1 (2)	O4'—C7'—C8'—C15'	−22.4 (3)
O2—C7—C8—C15	160.11 (15)	O2'—C7'—C8'—C15'	163.40 (16)
O4—C7—C8—C14	82.8 (2)	O4'—C7'—C8'—C14'	85.9 (2)
O2—C7—C8—C14	−91.99 (19)	O2'—C7'—C8'—C14'	−88.37 (19)
O4—C7—C8—C9	−151.57 (17)	O4'—C7'—C8'—C9'	−150.20 (18)
O2—C7—C8—C9	33.6 (2)	O2'—C7'—C8'—C9'	35.6 (2)
C7—C8—C9—C11	−132.33 (16)	C7'—C8'—C9'—C11'	−134.01 (16)
C15—C8—C9—C11	101.34 (17)	C15'—C8'—C9'—C11'	98.59 (17)
C14—C8—C9—C11	−8.5 (2)	C14'—C8'—C9'—C11'	−12.5 (2)
C7—C8—C9—C10	−5.4 (2)	C7'—C8'—C9'—C10'	−7.7 (2)
C15—C8—C9—C10	−131.72 (15)	C15'—C8'—C9'—C10'	−135.12 (15)
C14—C8—C9—C10	118.45 (16)	C14'—C8'—C9'—C10'	113.84 (16)
O2—C1—C10—C9	70.23 (17)	O2'—C1'—C10'—C20'	−49.3 (2)
C2—C1—C10—C9	−168.21 (15)	C2'—C1'—C10'—C20'	71.7 (2)
O2—C1—C10—C5	−164.51 (14)	O2'—C1'—C10'—C5'	−163.28 (14)
C2—C1—C10—C5	−42.9 (2)	C2'—C1'—C10'—C5'	−42.3 (2)
O2—C1—C10—C20	−51.2 (2)	O2'—C1'—C10'—C9'	70.76 (17)
C2—C1—C10—C20	70.4 (2)	C2'—C1'—C10'—C9'	−168.20 (15)
C11—C9—C10—C1	83.26 (17)	C6'—C5'—C10'—C1'	159.79 (15)
C8—C9—C10—C1	−42.23 (18)	C4'—C5'—C10'—C1'	36.3 (2)
C11—C9—C10—C5	−41.6 (2)	C6'—C5'—C10'—C20'	38.96 (17)
C8—C9—C10—C5	−167.09 (14)	C4'—C5'—C10'—C20'	−84.50 (18)
C11—C9—C10—C20	−153.49 (15)	C6'—C5'—C10'—C9'	−78.50 (17)
C8—C9—C10—C20	81.02 (17)	C4'—C5'—C10'—C9'	158.05 (15)
C6—C5—C10—C1	161.27 (15)	C11'—C9'—C10'—C1'	83.32 (18)
C4—C5—C10—C1	37.1 (2)	C8'—C9'—C10'—C1'	−41.29 (18)
C6—C5—C10—C9	−77.59 (17)	C11'—C9'—C10'—C20'	−154.39 (15)
C4—C5—C10—C9	158.22 (15)	C8'—C9'—C10'—C20'	81.00 (18)
C6—C5—C10—C20	40.27 (16)	C11'—C9'—C10'—C5'	−41.7 (2)
C4—C5—C10—C20	−83.92 (18)	C8'—C9'—C10'—C5'	−166.34 (15)
C10—C9—C11—C12	−171.65 (15)	C10'—C9'—C11'—C12'	−170.93 (15)
C8—C9—C11—C12	−45.8 (2)	C8'—C9'—C11'—C12'	−46.0 (2)
C9—C11—C12—C13	39.6 (2)	C9'—C11'—C12'—C13'	46.8 (2)
C11—C12—C13—C16	−89.7 (2)	C11'—C12'—C13'—C16'	−97.08 (18)
C11—C12—C13—C14	21.6 (2)	C11'—C12'—C13'—C14'	13.1 (2)
C16—C13—C14—C8	44.46 (17)	C16'—C13'—C14'—C8'	47.58 (17)
C12—C13—C14—C8	−73.24 (17)	C12'—C13'—C14'—C8'	−68.75 (17)
C7—C8—C14—C13	−165.39 (15)	C7'—C8'—C14'—C13'	−163.99 (15)
C15—C8—C14—C13	−50.28 (16)	C15'—C8'—C14'—C13'	−48.65 (16)
C9—C8—C14—C13	66.42 (17)	C9'—C8'—C14'—C13'	69.34 (17)
C7—C8—C15—O5	−86.24 (18)	C7'—C8'—C15'—O5'	−93.50 (18)
C14—C8—C15—O5	160.42 (14)	C14'—C8'—C15'—O5'	154.24 (14)
C9—C8—C15—O5	43.12 (19)	C9'—C8'—C15'—O5'	36.5 (2)
C7—C8—C15—C16	149.71 (15)	C7'—C8'—C15'—C16'	142.94 (15)
C14—C8—C15—C16	36.37 (17)	C14'—C8'—C15'—C16'	30.68 (17)
C9—C8—C15—C16	−80.93 (17)	C9'—C8'—C15'—C16'	−87.02 (17)
C14—C13—C16—C17	157.1 (2)	C14'—C13'—C16'—C17'	147.5 (2)
C12—C13—C16—C17	−87.1 (2)	C12'—C13'—C16'—C17'	−96.8 (2)
C14—C13—C16—C15	−21.86 (19)	C14'—C13'—C16'—C15'	−28.65 (19)
C12—C13—C16—C15	93.90 (18)	C12'—C13'—C16'—C15'	86.99 (18)
O5—C15—C16—C17	50.6 (3)	O5'—C15'—C16'—C17'	60.7 (3)
C8—C15—C16—C17	171.54 (19)	C8'—C15'—C16'—C17'	−177.91 (19)
O5—C15—C16—C13	−130.43 (17)	O5'—C15'—C16'—C13'	−123.06 (17)
C8—C15—C16—C13	−9.46 (19)	C8'—C15'—C16'—C13'	−1.6 (2)
C6—O1—C20—O3	142.68 (15)	C6'—O1'—C20'—O3'	137.92 (15)
C6—O1—C20—C10	14.04 (19)	C6'—O1'—C20'—C10'	10.58 (19)
C1—C10—C20—O3	82.1 (2)	C1'—C10'—C20'—O3'	86.5 (2)
C9—C10—C20—O3	−36.9 (2)	C5'—C10'—C20'—O3'	−152.74 (16)
C5—C10—C20—O3	−157.09 (15)	C9'—C10'—C20'—O3'	−31.9 (2)
C1—C10—C20—O1	−154.68 (15)	C1'—C10'—C20'—O1'	−151.57 (15)
C9—C10—C20—O1	86.31 (17)	C5'—C10'—C20'—O1'	−30.85 (17)
C5—C10—C20—O1	−33.90 (17)	C9'—C10'—C20'—O1'	90.02 (17)

### Hydrogen-bond geometry (Å, °)

**Table:**

*D*—H···*A*	*D*—H	H···*A*	*D*···*A*	*D*—H···*A*
O3—H30···O5	0.85 (3)	1.88 (3)	2.695 (2)	161 (2)
O3'—H30'···O5'	0.88 (3)	2.01 (3)	2.806 (2)	150 (3)
O5—H50···O4'^i^	0.83 (3)	1.84 (3)	2.663 (2)	169 (3)
O5'—H50'···O1'^ii^	0.90 (3)	1.89 (3)	2.7663 (19)	165 (3)

Symmetry codes: (i) −*x*+1, *y*+1/2, −*z*; (ii) −*x*+1, *y*−1/2, −*z*.
